# Standardized Approach to Life History Data Collection in Poeciliid Fishes

**DOI:** 10.1002/ece3.70735

**Published:** 2025-02-19

**Authors:** Erik S. Johnson

**Affiliations:** ^1^ Department of Biology University of Missouri St. Louis Missouri USA; ^2^ Whitney R. Harris World Ecology Center St. Louis Missouri USA

**Keywords:** data collection, life history, livebearing fishes, Poeciliidae, standardized approach

## Abstract

Livebearing fishes in the family Poeciliidae have been essential to testing life history theory. These species are remarkable because males internally inseminate females, and females give birth to free‐swimming young, making these fishes amenable to investigating the evolution of a variety of life history traits, including the timing and nature of maternal reproductive investment, timing of maturity, strategies for maternal provisioning of embryos, and several other classic life history traits. However, researchers vary in the methods that they use to measure these traits, making it difficult to compare findings across studies. Here, I present a standardized approach to studying life history traits in livebearing fishes. I describe methods for preserving samples in the field, for collecting data on a standard set of life history traits, and for processing data in ways that will allow comparisons among studies. I highlight different options in preservation techniques and in data collection that are dependent on the specific questions being addressed. Finally, I argue for a standard approach moving forward to make it possible to complete large‐scale comparative studies to reveal how life history traits have evolved in this important model system.

## Graphical abstract

Research on livebearing fishes has been instrumental in the development of several elements of life history theory through empirical studies. We posit that a standardized approach to life history data collection within this singular system is needed to address the current important questions in life history evolution through large comparative studies.

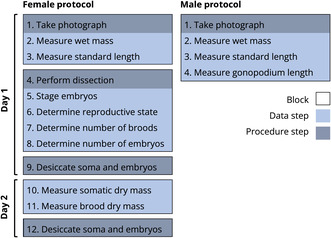

## 1 Introduction

Ever since Darwin, biologists have tested predictions of evolutionary theory against empirical and experimental findings. Perhaps no field has enjoyed such a rich blending of theoretical expectations with empirical data as the study of life history evolution (Roff 1993, 2002; Stearns 1992). Indeed, understanding how organisms adjust their reproductive schedules throughout their lives in response to different selective agents has provided powerful insights into the tempo and mode of evolution in natural systems (Caswell 1983; Gotthard and Nylin 1995; Nylin and Gotthard 1998; Potter et al. 2021; Salguero‐Gómez and Jones 2017), how evolutionary diversification occurs (Day, Abrams, and Chase 2002; Derrickson and Ricklefs 1988; Owens and Bennett 1995; Ricklefs 2000; White et al. 2013; Winemiller and Rose 1992), and the extent to which evolution is predictable and repeatable in the wild (Johnson and Belk 2001; Moore, Riesch, and Martin 2016; D. N. Reznick, Rodd, and Cardenas 1996; Riesch et al. 2014).

Interestingly, much of the progress in understanding life history evolution has relied heavily on a relatively small number of model systems for which life history data can be readily collected, including *Arabidopsis* plants (Alonso‐Blanco et al. 1999; Donohue 2002; Ellis et al. 2021), Great Tit birds (Cole et al. 2012; Lack 1954; Wilkin et al. 2006), *Drosophila* fruit flies (Schmidt et al. 2005; Schwarzkopf, Blows, and Caley 1999; Sgrò and Partridge 2000), and others (Berry and Bronson 1992; Bongers 1999; Harvey, Shorto, and Viney 2008; Svihla 1933). Yet, among all model systems, livebearing fishes in the family Poeciliidae have had a disproportionate impact as empirical models to test life history theory (Alcaraz and García‐Berthou 2007; Bono, Rios‐Cardenas, and Morris 2011; Downhower, Brown, and Matsui 2000; Evans, Pilastro, and Schlupp 2011; Frías‐Alvarez et al. 2014; Golden, Belk, and Johnson 2021; Hulthén et al. 2021; Jaime Zúñiga‐Vega, Reznick, and Johnson 2007; Johnson and Belk 2001; José, Rodríguez, and León 2013; Mukherjee et al. 2014; Pollux et al. 2009; Reznick, Rodd, and Cardenas 1996; Reznick 1997; Reznick and Bryga 1996; Riesch, Martin, and Langerhans 2013, 2020; Riesch et al. 2014; Roth‐Monzón et al. 2021; Schlupp, Taebel‐Hellwig, and Tobler 2010; Weldele, Jaime Zúñiga‐Vega, and Johnson 2014; Zúñiga‐Vega et al. 2024). Pioneering work done in the Trinidadian guppy ( *Poecilia reticulata* ) (Reznick et al. 2001; Reznick and Endler 1982; Reznick 1997) has inspired dozens of additional research studies in several other livebearing fishes, each testing some aspect of life history theory. In fact, we are fast approaching the point where large‐scale comparative studies across the almost 300 species of Poeciliid fishes are likely to yield important insights into how reproductive traits evolve in livebearing organisms.

Given the potential to understand life history evolution in a large‐scale comparative framework, it is critical that the data evaluated be comparable among species and across different studies. How life history data have been collected in livebearing fishes has mostly been passed along by word‐of‐mouth and by duplicating methods in the literature for the past several decades, resulting in similarities and also some important differences among published studies. Understanding these varied methodological approaches is important for interpreting past studies and will be essential for guiding future work. However, a uniform approach to data collection could ensure more meaningful comparative analyses. To this end, what is needed is a careful description of various techniques that have been employed in life history research for livebearing fishes and a standardized framework for collecting and processing data moving forward.

Here, I do just that. I provide a brief background on collecting life history data from livebearing fishes, beginning with how specimens are collected and preserved in the field. I then describe a step‐by‐step technique for processing fish specimens to collect data for each of several life history traits. I show how a standard workflow facilitates data collection and preserves the specimen and its constituent parts for future study. I describe differences in this process as presented in different research publications, some of which could make it difficult to compare studies. Finally, I suggest when different approaches should be deployed for different research questions, and I argue for a uniform approach to collecting life history data moving forward so that comparative studies can be effectively completed.

## 2 Life History Data Collection

### 2.1 Field Sampling and Specimen Preservation

How specimens are sampled and preserved can have an important impact on the life history traits that can be evaluated. Adequate sample sizes are necessary to quantify certain life history traits, as is sampling across the entire size distribution of fish in a population. Hence, a carefully designed approach to both field sampling and specimen preservation techniques is essential.

#### 2.1.1 Field Sampling

Selecting sites from which to sample, of course, depends on the specific research question—previous life history studies in livebearers have focused on the effects of predation (Jennions and Telford 2002; Johnson 2001; Johnson and Zúñiga‐Vega 2009; Reznick 1982; Reznick, Bryga, and Endler 1990), density (Bassar et al. 2013; Johnson and Bagley 2011; Reznick et al. 2012), seasonality (Johnson, Tobler, and Johnson 2023; Winemiller 1993), and stream gradient (Jaime Zúñiga‐Vega, Reznick, and Johnson 2007), all evaluated as agents of natural selection. Hence, in each of these studies, it is necessary to sample populations from sites with contrasting selective regimes. However, regardless of the specific question, all life history studies should have a common standard for sampling at a particular site. First, it is critical that a sampling site have enough individuals to characterize the life history. Unlike other types of studies that require fewer individuals (e.g., stable isotopes, behavior, and morphometrics), life history studies require a relatively large sample size. In general, a minimum of 70 individuals—30 mature females, 20 mature males, and 20 juveniles—is typically needed to draw robust conclusions about life history traits within a population. Mature fish should be sampled from across the size distribution of individuals within a category. Ideally, fish should be collected randomly from a particular site to represent the full range of variation among individuals. It is important to avoid exhaustive sampling (i.e., repeated seining from one pool or one reach of a stream) to achieve adequate sample sizes, both for conservation purposes and to avoid sampling bias within a collection. Second, when collecting fish from populations in the wild, it is important to recognize that each site can be ecologically distinct. Hence, in addition to collecting fish, researchers should gather data on several ecological variables that can covary with certain life history traits. Quantifying the density and abundance of aquatic predator species (Gorini‐Pacheco, Zandonà, and Mazzoni 2018; Johnson 2001; Johnson and Zúñiga‐Vega 2009; Reznick 1982; Reznick, Bryga, and Endler 1990), the presence of potential competitors (Scott and Johnson 2010), the season in which a sample is taken (Johnson, Tobler, and Johnson 2023; Reznick 1989a), estimates of canopy cover or resource availability (Grether et al. 2001; Walsh and Reznick 2009), water temperature and water chemistry (Martin et al. 2009; McManus and Travis 1998; Riesch, Plath, and Schlupp 2010), and stream gradient (Jaime Zúñiga‐Vega, Reznick, and Johnson 2007) are all ecological factors that should be considered when trying to understand variation among sites in life history traits.

#### 2.1.2 Specimen Collection and Preservation

Once a fish is collected, it must be preserved so that it can later be used to provide life history data in the laboratory. Collection techniques often vary depending on characteristics of the riverine environment, including substrate type, the presence of boulders or emergent vegetation, the size of the river, etc. Fine‐mesh dip nets and cast nets have both been used in this type of sampling. However, the most common method to collect livebearing fishes is the use of a handheld seine. Following collection, live fish can be held in a tank or bucket to ensure that adequate numbers of specimens will be collected at a particular site. If not, live fish should be returned to the river. If adequate numbers can be achieved, specimens should be euthanized and then fixed in a preservative solution. The ASIH guide for field collections (Nickum 1988) recommends using an overdose of tricaine methane sulfonate (MS222) or rapid cooling of fish in ice as appropriate methods to euthanize individuals.

The researcher must choose one of two methods to preserve specimens: (1) fixation in 100% ethanol or (2) fixation in 40% buffered formalin. Fish fixed in formalin retain the mass of lipid material in the specimen, including in the eggs, whereas fish fixed in ethanol lose their fat content, which is dissolved into the alcohol solution. Each of these techniques has pros and cons, and choosing one or the other depends on the research question asked (as described below). Preserved specimens are then transported to the laboratory where life history data will be collected.

### 2.2 Laboratory Specimen Processing

**FIGURE 1:**
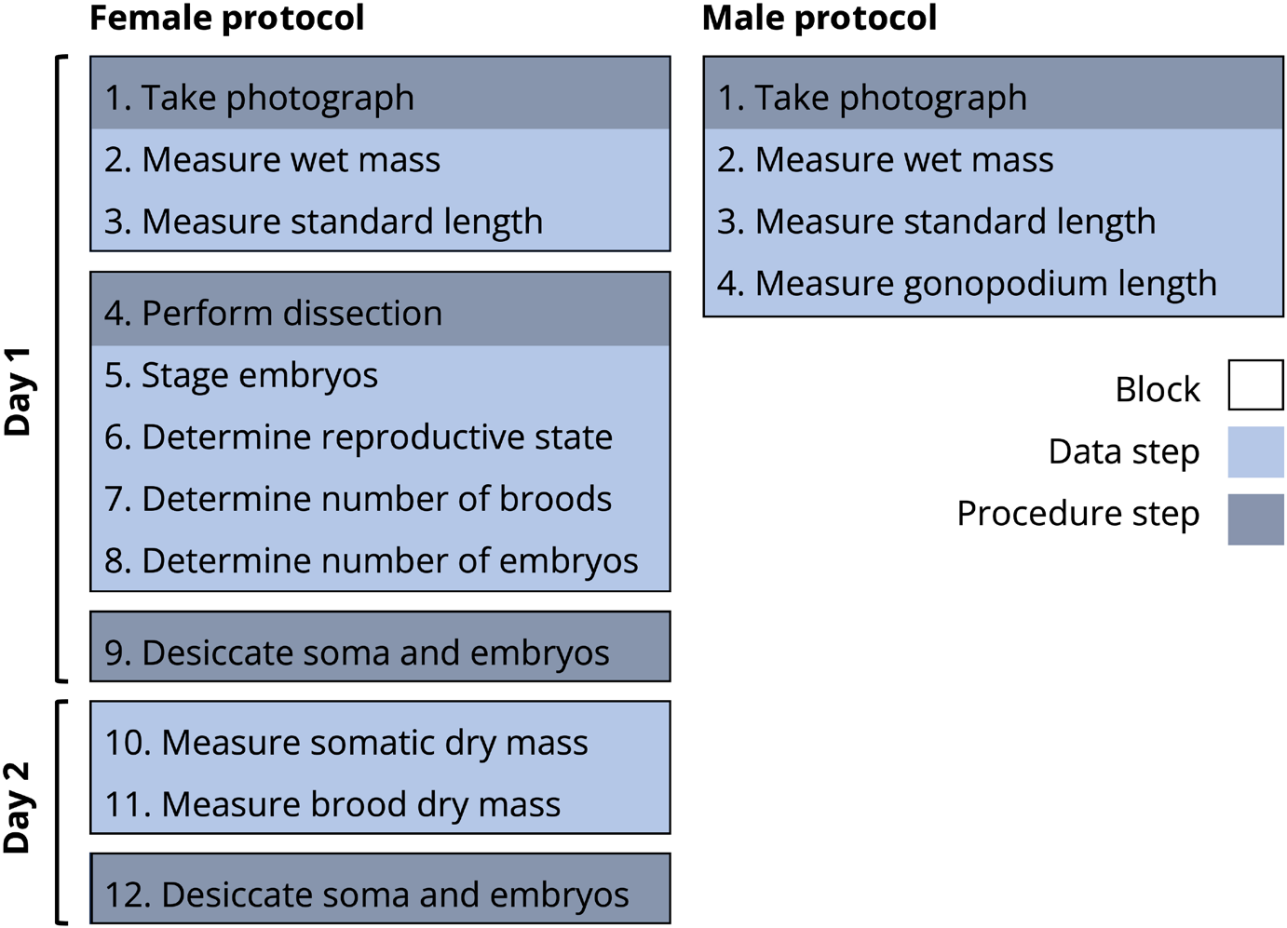
Standard workflow designed to collect life history data from poeciliid fishes for females (left column) and males (right column). This workflow spans 2 days to allow for a 24‐h period for specimens to desiccate in a drying oven. The workflow begins on top and progresses down from step to step. “Data step” refers to each step where data are collected. “Procedure step” refers to any step that is required to manipulate specimens for data collection or preservation. “Block” refers to a group of steps that are most efficient when performed on all individuals consecutively before continuing to the next block.

The first step in processing specimens (Figure 1) is to separate adult males from the remaining specimens. Adult males are distinguished from juveniles by the presence of a fully developed gonopodium, the male intromittent organ. In most species, it is also possible to identify juvenile males by a gonopodium present that is not fully developed, and in some cases juvenile males can be identified by the thickening of the third fin ray on the anal fin (Greven 2011); to see the third fin ray clearly under a microscope might require removing soft tissue from the surface of the gonopodium. This initial sorting leaves the researcher with two groups of male specimens (mature and immature) and a group that includes both adult females and juveniles (including both indistinguishable immature males and immature females). From these three groups of specimens, data on all life history traits (Box 1) for both males and females can be collected. Here, I detail how these life history trait data are collected—first describing data collected from all specimens and then data uniquely collected from each sex.

BOX 1 **Data collected from specimens.**

*All specimens*

– Standard length
– Wet mass

*Males*

– Reproductive state
– Size at maturity
– Gonopodium length

*Females*

– Reproductive state
– Number of broods
– Number of offspring per brood
– Size of offspring
– Embryo stage
– Brood dry mass
– Somatic dry mass

#### 2.2.1 Data to Collect From Each Specimen

The process of data collection is different for males and females, but several kinds of life history data should be gathered initially from all specimens, including standard length and wet mass (Box 1, details described below). Additionally, if specimens are used for other analyses such as color, shape, or functional morphology, photographs should be taken at this time. Best colors occur in live fish prior to preservation, although in formalin some color remains for a short period of time following fixation. Photographs for these analyses are typically taken with a lateral view, with the head to the left to expose the left flank.

#### 2.2.2 Male Data Collection Protocol

**FIGURE 2:**
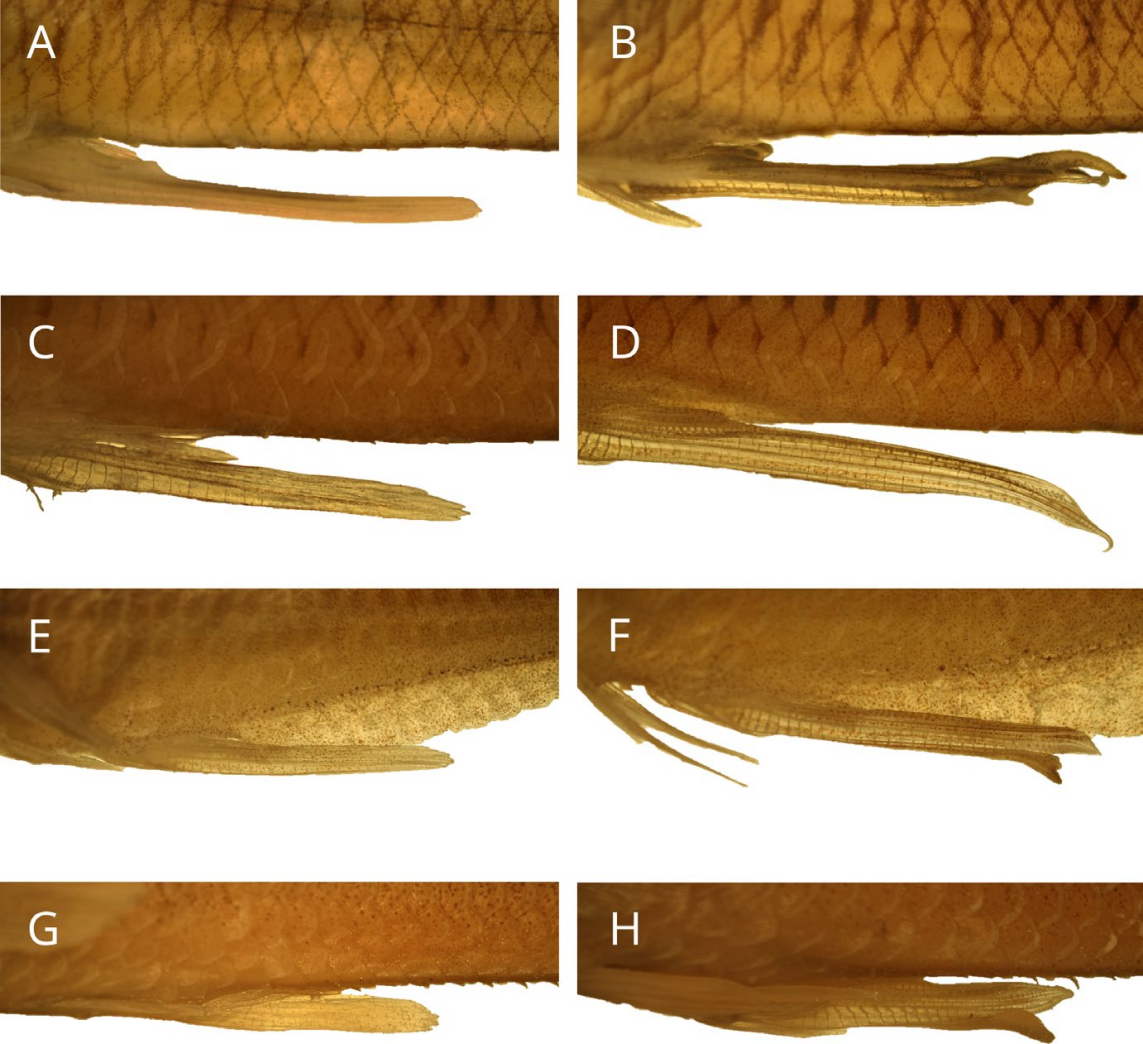
Images of male gonopodia contrasting juvenile and adult males in four poeciliid fish species: *Xenophallus umbratilis* (A, B); *Pseudoxiphophorus bimaculatus* (C, D); *Alfaro cultratus* (E, F); *Poecilia mexicana* (G, H).

**FIGURE 3:**
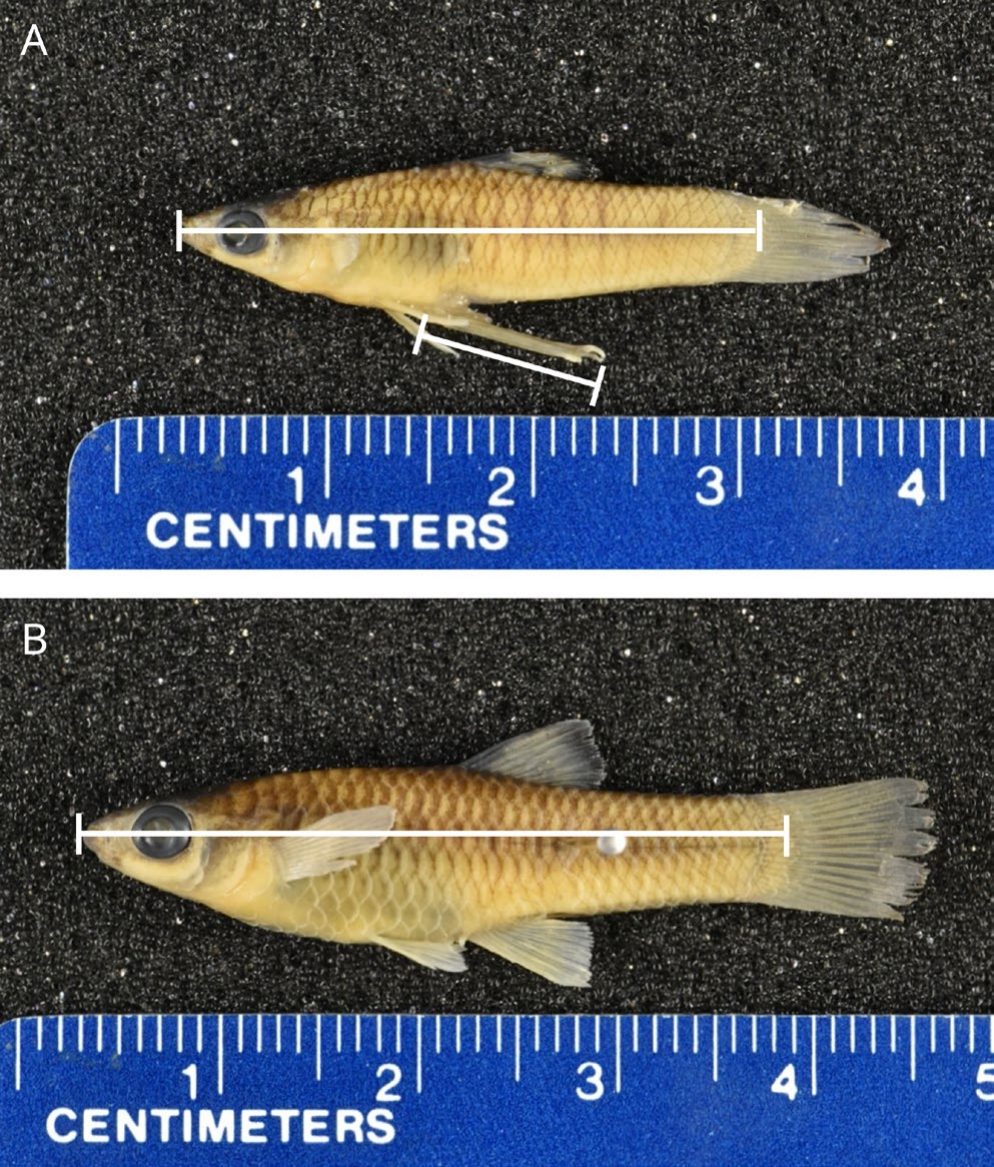
(A) Image of a male poeciliid, *Xenophallus umbratilis*, showing the measurements of standard length and gonopodium length. (B) Image of a female poeciliid, *Xenophallus umbratilis*, showing measurement of standard length (see text for description).

1. To determine **reproductive state**, classify males as mature or immature by examining the anal fin/gonopodium. A mature male is classified by the presence of a fully developed gonopodium, which in many species is marked by the presence of hooks, barbs, serrae, or fleshy palps; however, gonopodium structure is unique to each species (Langerhans 2011). As a male matures, the appearance of the gonopodium typically changes from a cloudy appearance to a clear and translucent appearance (Figure 2). Regardless of structure or appearance, until the gonopodium is completely developed, an individual male cannot successfully transfer sperm to a female (Greven 2011).
2. Use a balance to measure the **wet mass** of the specimen. This measure is taken after all excess liquid has been removed from the surface of the fish, typically by using absorbent tissue, such as ChemWipes or a paper towel.
3. Use calipers to measure the **standard length**, defined as the distance from the tip of the snout to the terminus of the vertebral column, readily seen as the point where the musculature anterior to the caudal fin tapers (Figure 3A).
4. Use calipers to measure the **gonopodium length**, defined as the distance from the tip of the gonopodium to the base of the gonopodium, the point where it extends from the body (Figure 3A).

No additional male life history data are typically collected beyond these. However, it is possible to measure the mass of male gonads (testes) in fresh specimens or specimens that have been preserved in formalin (Schlupp, Poschadel, and Tobler 2006; Schrader et al. 2012). This is done by dissection, removing the testes from the body and weighing the gonads in either wet or dry form (following Schlupp, Poschadel, and Tobler 2006 and Schrader et al. 2012). However, this step is optional given that life history studies seldom focus on male reproductive investment—more often, testes mass is measured for studies of sperm competition (Schrader et al. 2012).

#### 2.2.3 Female Data Collection Protocol

1. Use a balance to measure the **wet mass** of the specimen. Prior to weighing, dab the fish dry using a paper towel or ChemWipes until all excess alcohol is removed from the sample.
2. Use calipers to measure the **standard length** of the individual (Figure 3B).
3. Dissect the female and remove the digestive tract and embryos (if present). This is done using small dissection scissors. Make a mid‐sagittal cut along the ventral surface of the fish. Only cut through the body wall and avoid cutting any organs within the body cavity, including the GI tract. Initiate the cut directly anterior to the insertion of the anal fin and cut anteriorly to a point directly between the insertions of the pectoral fins. On the left side of the fish, beginning where the ventral cut ended, cut dorsally to a point even with the lateral line (approximately 2/3 of the way up the body). Make sure to cut under the pectoral fin to avoid cutting through this structure. Still on the left side, make a second vertical cut dorsally beginning from the point of the first incision, and continue up to a point even with the lateral line (again, approximately 2/3 of the way up the body). These three cuts should form a flap on the left side of the fish. Fold this flap upward (dorsally), exposing the contents of the body cavity. Break the ribs of the left side by rotating the flap upward using a pair of forceps—this will open the flap and reveal a window into the body cavity. Once the window is open and the body cavity is exposed, identify the digestive tract. Use forceps to remove the digestive tract at the anus and at the anterior end of the body cavity. Preserve the digestive tract and its contents (if desired) by placing it into a vial filled with 70% ethanol. Then use forceps to remove embryos from the body cavity—embryos can often be removed in a single "sac" composed of the single ovary in these fishes. However, if the membrane around this sac is broken, the embryos must be removed one by one. These can be placed on the specimen plate of the dissecting scope. After embryos are removed, refold the flap of the female back into its natural closed position. Place the specimen into a plastic weigh boat, now ready to be dried.
4. Determine the **developmental stage of the embryos** by viewing them underneath a dissection scope. Embryos have historically been staged using either the Reznick method (Reznick 1981; Reznick, Mateos, and Springer 2002) or the Haynes method (Haynes 1995)—I compare these two approaches below.
5. Determine the **reproductive state** of each female specimen. A mature female is classified by the presence of fertilized, developing embryos, or by the presence of fully yolked eggs. Females with no embryos or unfertilized embryos are classified as immature.
6. Determine the **number of developing broods** by observing whether multiple stages of development are present among embryos within one specimen (excluding unfertilized ova). The phenomenon of carrying more than one brood at different developmental stages simultaneously in a single individual is known as superfetation (Scrimshaw 1944). Fish that carry only one brood at a time are defined as non‐superfetating. In some non‐superfetating species, unfertilized eggs that are yolked are retained while fertilized embryos develop—these unfertilized eggs should not be mistaken for, or counted as, developing embryos.
7. Count and record the total **number of embryos** in each developmental stage. Place these embryos into a plastic weigh boat.
8. Place both the female and her embryos, both now on weigh boats, into a drying oven set to 50°C and allow these specimens to dry for at least 24 h, after which no additional desiccation occurs. However, drying a specimen for more than 48 h can result in damage to the specimen.
9. Remove specimens from the drying oven. Immediately measure the **somatic dry mass** and **brood dry mass** using a balance. It is important to take these measurements immediately, as humidity in the air will cause specimens to rehydrate (at different rates depending on the humidity), which is seen as a very minute increase in weight as the specimen sits on the balance.
10. Finally, rehydrate specimens using distilled or deionized (DI) water. To rehydrate, place the specimen in a weigh boat filled with water for approximately 10–20 min, depending on the size of the fish. Adequately rehydrated samples will be flexible and no longer brittle. At this point, identification tags can be attached using a needle and museum‐grade string. Samples can then be returned to a jar of 70% ethanol for long‐term storage.

After these steps are complete, the researcher will have gathered all life history trait data that are typically collected in any life history study.

## 3 Life History Data Processing

After collecting the necessary data using the protocols above, data processing, including some simple calculations, is necessary to quantify some life history traits prior to analysis. I describe how this data processing is accomplished for the six life history traits where this is necessary (Box 2).

### 3.1 Male Size at Maturity

Male size at maturity within populations is calculated by taking the mean standard length value among all mature males within the population. Given that males cease growth upon maturation, the mean body size is frequently used to report size at maturity (Johnson and Bagley 2011). Additionally, some researchers choose to report the distribution of male standard lengths, which can reveal the presence of alternative male mating strategies (Cohen et al. 2015; Furness, Hagmayer, et al. 2020). It can also be helpful to examine the size range of maturing males—in some cases, the size of immature males can overlap with or exceed the size of mature males (Johnson and Bagley 2011).

BOX 2 **Traits calculated from raw data.**

– Male size at maturity
– Female size at maturity
– Maternal resource provisioning strategy (lecithotrophy or matrotrophy)
– Female reproductive allotment/somatic investment
– Fecundity
– Superfetation

### 3.2 Female Size at Maturity

**FIGURE 4:**
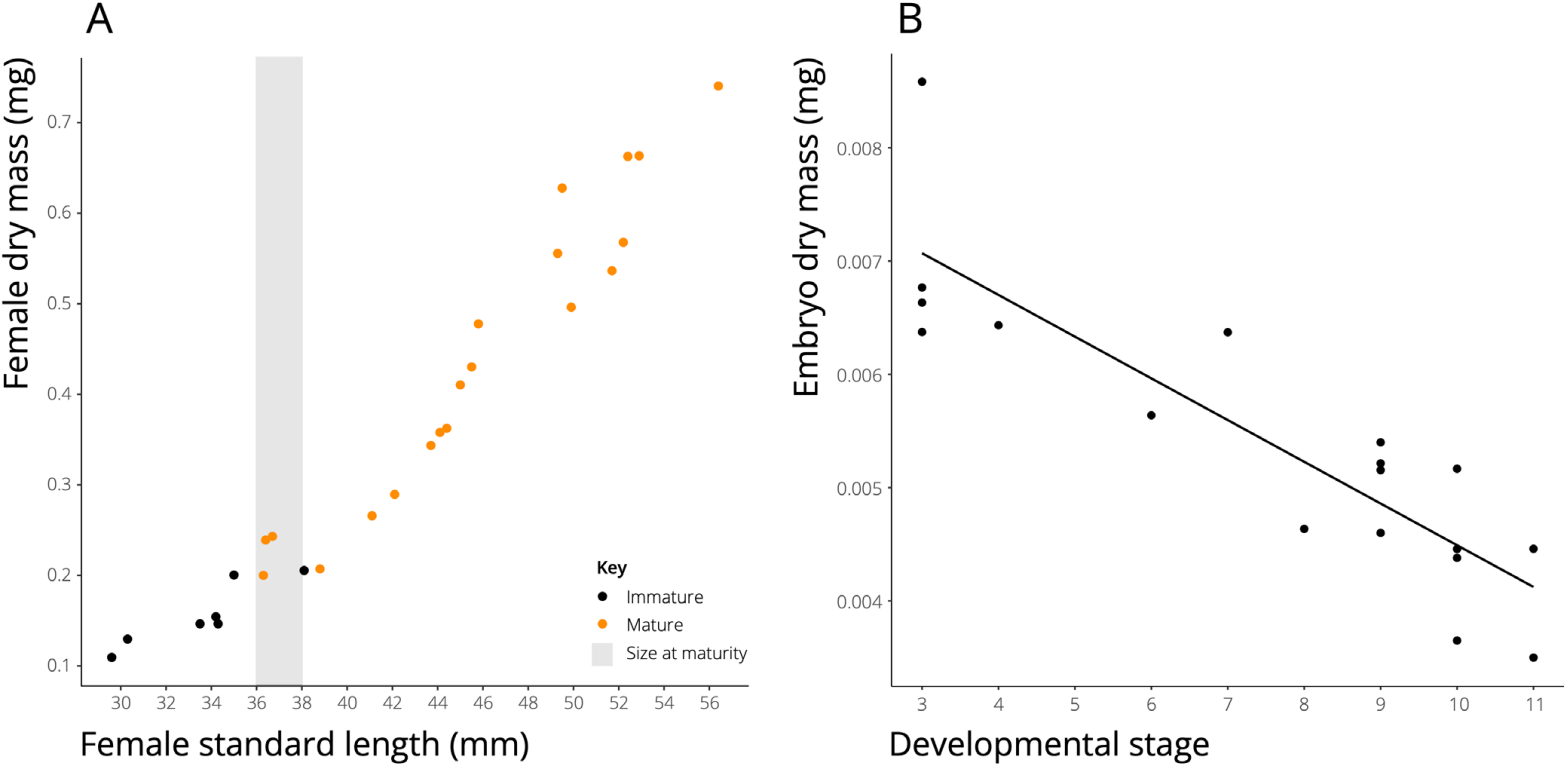
(A) Plot showing the relationship between somatic dry mass of females and standard length of females in a population of livebearing fish. Data points represent individual fish, with immature individuals marked in black and mature individuals marked in orange. The 2 mm size class in which most individuals are mature is marked by a vertical gray bar, in this case the 36‐38 mm size class. Data presented were collected from *Priapichthys annectens* at Rio Colorado in Guanacaste, Costa Rica (Johnson, Tobler, and Johnson 2023). (B) Plot showing the relationship between embryo mass and embryo developmental stage (following the Haynes method; see text). The slope of this line is used to describe the maternal resource provisioning strategy, with positive and flat slopes indicating maternal provisioning throughout embryonic development (i.e., matrotrophy) and a negative slope indicating no maternal provisioning after the egg is fertilized (i.e., lecithotrophy). The data presented here come from *Priapichthys annectens* taken from a tributary to Rio Cucaracho in Guanacaste, Costa Rica (Johnson, Tobler, and Johnson 2023).

Female size at maturity within each population is calculated as follows. Females are typically divided by standard length into 2 mm length classes. However, smaller size classes might be preferred (e.g., 1 mm size classes) if the sample size is sufficiently large or if the average body size of the species is particularly small. The size at maturity is then defined as the smallest length class in which at least half of individuals are mature. I provide a plot of female dry mass and standard length to visually illustrate this (Figure 4A). Confidence in the size and maturity estimate for females from a population is greatest when all size classes below the determined size at maturity are composed of immature individuals, and all size classes above the determined size at maturity are composed of mature individuals. There may be, however, some exceptions to this pattern in temperate species that reproduce seasonally (Reznick and Braun 1987) where mature females that are out‐of‐season are not carrying developing embryos. Even in tropical environments, some species slow or cease reproduction between wet and dry seasons (Chapman, Kramer, and Chapman 1991; Chapman and Chapman 1993; Reznick 1989b). These phenomena may bias estimates of female size at maturity. One indicator that a species is not reproducing year‐round is the presence of females in larger size classes that are found without developing embryos.

### 3.3 Maternal Resource Provisioning Strategy

In livebearing fishes, females provision their developing embryos either by loading their eggs entirely with nutrients prior to fertilization (lecithotrophy) or by provisioning their fertilized embryos with nutrients throughout embryonic development (matrotrophy). Most livebearing fishes reproduce year‐round; however, in some species, reproduction can be seasonal, ceasing during the winter or the dry season (Winemiller 1993). In nature, the degree of maternal provisioning can vary along a gradient from complete lecithotrophy to extreme matrotrophy (Pollux et al. 2009). This trait is measured by examining the relationship between the stage of development of embryos (see below how this is defined) and the mass of an individual embryo. Individual embryo mass is calculated by dividing brood dry mass by the number of embryos in the brood. Plotting the relationship between individual embryo mass and developmental stage reveals if females are provisioning their developing offspring with nutrients throughout development or prior to development. A positive slope between these two variables—showing that embryo mass increases throughout embryonic development—is evidence for a matrotrophic provisioning strategy. Similarly, a zero slope also demonstrates that females are provisioning their young throughout development. Only when the slope is negative, where embryo mass decreases throughout development, is there evidence for lecithotrophy. To illustrate this, I provide a plot of individual embryo mass against developmental stage to visually demonstrate what this looks like for a lecithotrophic species (Figure 4B). The slope of the relationship between embryo mass and developmental stage has been defined as a "matrotrophy index" (Pollux et al. 2009).

### 3.4 Female Reproductive Allotment/Somatic Investment

Reproductive allotment is defined as the total biomass invested in reproduction, including the mass of all embryos and eggs. However, because larger females have the capacity for greater reproductive investment, this trait is often calibrated based on female body mass (so‐called "somatic mass," which is the mass of the female with both the GI tract and the reproductive tissues and embryos removed). Since reproductive allotment is often simply represented by the total brood dry mass, female "somatic mass" of the dried individual is often used as a random variable in models to control for size variation. Additionally, embryo stage should be included as a random variable as well, as embryo mass can change across developmental stages. In other cases, reproductive allotment is measured as a fraction of total mass (brood dry mass/brood dry mass plus somatic dry mass)—however, treating this ratio (sometimes called a gonadosomatic index) as a response variable can be problematic (see below), and most researchers opt for using somatic mass simply as a covariate.

### 3.5 Superfetation

In some species of livebearing fishes, females can simultaneously carry two or more developing broods fertilized at different times and are therefore at different developmental stages. This phenomenon is known as superfetation (Scrimshaw 1944). The number of broods in superfetating species can range from two up to as many as seven (e.g., in the highly superfetating least killifish, *Heterandria formosa* ; Guzmán‐Bárcenas and Uribe 2019). Detecting superfetation requires careful discernment to identify the presence of embryos at different developmental stages within a female. It is important to note that in non‐superfetating species, a single brood may appear to have embryos at slightly different developmental stages, but these are in fact fertilized at the same time. Additionally, in non‐superfetating species, unfertilized eggs can be present in the same tissue as fertilized, developing embryos. The presence of these unfertilized eggs should not be interpreted as superfetation.

### 3.6 Fecundity

Fecundity is simply the number of offspring produced. In non‐superfetating species, fecundity is measured by counting the number of embryos that are fertilized and undergoing development. In superfetating species, fecundity is usually defined by the number of embryos in the most developmentally advanced brood present. Some studies also count the total number of embryos across all broods, regardless of developmental stage. In some cases, fertilized embryos are spontaneously aborted, a process known as embryonic regression. These embryos should not be counted in fecundity measurements. Regressing embryos are usually found in less advanced stages of development than the majority of embryos and are characterized by a glassy appearance, while healthy embryos appear more cloudy (Furness, Avise, et al. 2021; Greven 2011; Norazmi‐Lokman, Purser, and Patil 2016).

## 4 Toward a Standardized Approach

Although life history data collection in livebearing fishes is mostly consistent across studies, some variation does exist. These differences can have an important impact on how some life history traits are described. Here I highlight some of these differences, and I explore their consequences.

### 4.1 Specimen Preservation Techniques

Two methods of specimen preservation have been used in poeciliids to prepare fish samples for life history study—these are fixation in ethanol and fixation in buffered formalin. There are advantages and disadvantages to each of these methods, and the decision to use one or the other is predicated on the nature of the research question. The choice of preservation technique can affect the measured values of some life history traits. Preservation in ethanol is often used because it is safer than using formalin (which is a carcinogen) and more convenient (because long‐term storage of samples is typically in 70% ethanol). Ethanol fixation also preserves DNA so that it can be used in future genomic studies. In contrast, formalin damages DNA, potentially alters nucleotide base pair integrity, and promotes cross‐linking among DNA molecules, making it difficult to sequence DNA (Koshiba et al. 1993). However, fixing samples in ethanol does impact some life history traits—ethanol is a solvent for fat. Hence, fixing samples in ethanol results in most fats being removed from the specimen. Ethanol also dehydrates specimens, which can alter both internal and external structural traits. This, in turn, affects measurements of body mass for adult fish and embryos and of reproductive allotment. In essence, ethanol‐fixed fish provide information on lean mass, with fat content removed. In contrast, fats are not soluble in formalin. So, an advantage of formalin‐preserved fish is that the fat content of the specimen remains intact. Most life history studies have used fish fixed in ethanol, not formalin. Yet, both techniques can be found in the published literature (Johnson and Bagley 2011). I recommend preserving specimens in formalin for life history studies and then retaining a subsample of specimens or tissues for genetic studies using ethanol preservation. Researchers should be aware of the effects of different preservation techniques when comparing among collections.

### 4.2 Embryo Staging Scales

There are two methods of embryo staging for life history research: these are the Reznick method (Reznick 1981; Reznick, Mateos, and Springer 2002) and the Haynes method (Haynes 1995). Both approaches assign numbers to embryos to depict their stage of embryonic development. There are advantages and disadvantages to each method. The Reznick method was developed first (Reznick 1981). This method scores embryos on a scale of 0 to 50, with developmental stages clustered into six major categories (0 = no development, 10 = uneyed, 20 = early‐eyed, 30 = mid‐eyed, 40 = late‐eyed, and 50 = very late‐eyed). The Reznick method utilizes increments of 5 to depict broods with individuals spanning two developmental stages (e.g., 35 describes broods with both mid‐eyed and late‐eyed embryos). The Haynes method, developed later, defines 11 developmental stages, with stages 1 and 2 being small‐ and medium‐sized unfertilized ova, respectively. Stages 3–11 define a progression of development, with stage 3 being a fully‐yoked egg that is fertilized and stage 11 being a fully developed embryo just prior to parturition. Stages in between are marked by phenotypic features that can be identified under a dissecting microscope, with traits such as blastodisc development, fin development, pigmentation, eye size, and scale development all featuring in the rubric. In essence, the Haynes method provides slightly finer resolution in terms of embryonic traits, although some traits might be difficult to see in preserved specimens. Unlike the Reznick method, the Haynes method illustrates how developmental stages appear in both lecithotrophic and matrotrophic maternal provisioning strategies.

Both the Reznick method and the Haynes method attempt to categorize stages of embryonic development, but do so by scoring prominent developmental stages that are readily identified under a dissecting microscope. More detailed staging of embryonic development is possible (Mousavi and Patil 2022), but it requires freshly harvested embryos from live fish and more specialized microscopy, which is generally not feasible for field‐collected specimens or from specimens currently housed in natural history collections. Interestingly, both the Reznick and Haynes staging methods have been used to plot embryo mass against developmental stage to score the degree of maternal provisioning, and both methods show a linear relationship between embryo mass and embryo stage (Reznick and Endler 1982). As described above, this provides a basis for calculating the matrotrophy index (Pollux et al. 2009). However, a problem with both methods is that neither approach uses stages that are explicitly linked to time—that is, neither purports to use stages as a surrogate for developmental age. Indeed, it is likely that embryos pass relatively rapidly through some stages and spend more time in others. Unfortunately, such a calibration has not yet been demonstrated. There may be some advantage to developing a staging system where each stage is defined by a set period of time as embryos move through developmental stages, which would reveal a more accurate view of the pace of development and which could be useful for comparing among species (E. S. Johnson, unpublished data). The embryo stage is often used as a covariate for other life history traits, such as embryo size and degree of matrotrophy, where it is treated as a continuous variable, when in fact it may not function exactly this way. Although the Reznick method is perfectly adequate, most life history studies use the Haynes method, likely due to the graphical distinction it makes between lecithotrophic and matrotrophic species (Haynes 1995). Given the bias in published studies across species toward the Haynes method, this is likely to continue to be the favored approach. However, a time‐based approach that also depicts distinct stages of embryo development may ultimately be more informative.

### 4.3 Statistical Approaches to Data Processing

Each life history research question can be addressed with a specific statistical approach appropriate to the particular study. Prior to these analyses, as I have detailed above, researchers either directly measure a life history trait (e.g., number of offspring, number of broods, male size at maturity, etc.) or they calculate the life history trait (e.g., female size at maturity within a population, degree of matrotrophy, embryo size, etc.). How these latter trait values are calculated is important. Because many life history traits vary with body size, trait values must be size adjusted. Some researchers have proposed creating size‐adjusted trait values as response variables prior to conducting statistical analyses—for example, the gonadosomatic index (GSI) that is common in fisheries research. However, using ratios (such as the GSI) can present difficulties in general linear model analyses (Lien, Hu, and Liu 2017). That is, how life history traits vary as a function of size can differ ontogenetically (allometric scaling), and these relationships can also differ among populations (e.g., Johnson, Tobler, and Johnson 2023). Hence, most researchers treat covariates as independent factors in statistical models as part of the analyses. An important caveat in these analyses is that different life history traits can covary with covariates in different ways. For example, some work has shown that as females grow larger, not only do they have more offspring, but those offspring are smaller (Reznick and Endler 1982). In these cases, it may be necessary to compare life history trait values at specific female sizes rather than relying on an analysis of covariance where the model assumptions are not met.

## 5 Why a Standardized Approach? The Future of Life History Research in POECILIIDS

The use of poeciliids to test life history theory has an impressive history (Johnson and Bagley 2011). Yet, the stage is set for this group of fishes to provide new answers to new questions in life history research, especially questions that examine the evolution of life history strategies over time and space. Incorporating a standardized set of techniques to collect life history data will be critical to facilitate this work.

Comparative studies that examine how life history traits evolve through evolutionary time offer great promise. Recent phylogenetic work shows the relationships among most species within Poeciliidae (Furness et al. 2019; Rodríguez‐Machado et al. 2024), rendering the system particularly useful for comparative work. Indeed, some comparative research in poeciliids has already begun. Small‐scale studies comparing a few species already exist (Frías‐Alvarez et al. 2014; Plath et al. 2007; Swenton and Kodric‐Brown 2012), while genera‐wide studies are less common (but see work done in *Poecilia* (Pires and Reznick 2018), *Limia* (Cohen et al. 2015), and *Phallichthys* (Regus et al. 2013)). Family‐wide life history studies are few but appear to be very promising. For example, research on viviparity in livebearing fishes comparing multiple species across the entire family has revealed insights into the evolution of placentation (Furness et al. 2019; Furness, Avise, et al. 2021; Meredith et al. 2010), superfetation (Furness et al. 2019), and matrotrophy (Olivera‐Tlahuel et al. 2015). These types of studies are particularly adept at exploring if the presence of certain life history traits primes the evolution of subsequent traits (e.g., matrotrophy preceding superfetation). Family‐wide comparative studies might also yield additional insights into other aspects of life history evolution, including ontogenetic shifts in patterns of reproductive allotment, understanding how sexual conflict affects life histories, and basic insights into how male life histories evolve. Finally, comparisons among species across the phylogeny can also reveal evolutionary interactions between life history strategies and other traits known to vary among livebearing fishes, including alternative male mating strategies and functional morphological adaptations. While these interactions have been probed within some species (Domínguez‐Castanedo et al. 2023; Furness, Hagmayer, et al. 2021), understanding how life history strategies evolve over time, and in relation to the evolution of other traits, is likely only to be uncovered within a phylogenetic framework when multiple species are compared.

Ultimately, addressing any of these questions will require having comparable life history data for multiple species. Hence, adopting a standardized approach to collect, manipulate, and analyze data to score life history traits in livebearing fishes will allow this model system to continue to contribute to our understanding of life history theory now and into the future.

## Author Contributions

**Erik S. Johnson:** conceptualization (equal), data curation (equal), formal analysis (equal), funding acquisition (equal), investigation (equal), methodology (equal), project administration (equal), resources (equal), software (equal), supervision (equal), validation (equal), visualization (equal), writing – original draft (equal), writing – review and editing (equal).

## Conflicts of Interest

The author declares no conflicts of interest.

## Data Availability

All data presented in this paper are available either in the paper itself or in other referenced papers.
